# Detection and counting of *Leishmania* intracellular parasites in microscopy images

**DOI:** 10.3389/fmedt.2024.1360280

**Published:** 2024-08-23

**Authors:** Lariza María de la Caridad Portuondo-Mallet, Niurka Mollineda-Diogo, Rubén Orozco-Morales, Juan Valentín Lorenzo-Ginori

**Affiliations:** ^1^ Centro de Estudios de Neurociencias, Procesamiento de Imágenes y Señales (CENPIS), Universidad de Oriente, Santiago de Cuba, Cuba; ^2^ Centro de Investigaciones de la Informática (CII), Universidad Central “Marta Abreu” de Las Villas, Santa Clara, Cuba; ^3^ Centro de Bioactivos Químicos (CBQ), Universidad Central “Marta Abreu” de Las Villas, Santa Clara, Cuba; ^4^ Centro de Estudios de Mecánica Computacional y Métodos Numéricos en la Ingeniería (CIMCNI), Universidad Central “Marta Abreu” de Las Villas, Santa Clara, Cuba

**Keywords:** image segmentation, *Leishmania*, amastigote, multilevel Otsu, watershed segmentation

## Abstract

**Problem:**

Leishmaniasis is a disease caused by protozoan parasites of the genus *Leishmania* and has a high prevalence and impact on global health. Currently, the available drugs for its treatment have drawbacks, such as high toxicity, resistance of the parasite, and high cost. Therefore, the search for new, more effective, and safe drugs is a priority. The effectiveness of an anti-leishmanial drug is analyzed through *in vitro* studies in which a technician manually counts the intracellular form of the parasite (amastigote) within macrophages, which is slow, laborious, and prone to errors.

**Objective(s):**

To develop a computational system that facilitates the detection and counting of amastigotes in microscopy images obtained from *in vitro* studies using image processing techniques.

**Methodology:**

Segmentation of objects in the microscope image that might be *Leishmania* amastigotes was performed using the multilevel Otsu method on the saturation component of the *hue, saturation, and intensity* color model. In addition, morphological operations and the watershed transform combined with the weighted external distance transform were used to separate clustered objects. Then positive (amastigote) objects were detected (and consequently counted) using a classifier algorithm, the selection of which as well as the definition of the features to be used were also part of this research. MATLAB was used for the development of the system.

**Results and discussion:**

The results were evaluated in terms of sensitivity, precision, and the F-measure and suggested a favorable effectiveness of the proposed method.

**Conclusions:**

This system can help researchers by allowing large volumes of images of amastigotes to be counted using an automatic image analysis technique.

## 1 Introduction

Leishmaniasis is a disease caused by protozoan parasites of the genus *Leishmania* and continues to be a major public health problem in several tropical and subtropical countries. It is transmitted by the bite of sandfly, mainly *Phlebotomus* in Europe, Asia, and Africa, and *Lutzomyia* in America (1). According to the World Health Organization (WHO), leishmaniasis is among the top ten neglected tropical diseases, with an estimated 1.6 million new cases each year and between 20,000 and 30,000 deaths (2). There are three main forms of leishmaniasis: visceral (the most serious form because it is usually fatal without treatment), cutaneous (the most common, usually causing skin ulcers), and mucocutaneous (affecting the mouth, nose, and throat) (2). Currently, there is no available vaccine for this disease, and the drugs used for its treatment have serious limitations, such as parasite resistance, high cost, and strong side effects for the patient (3). Therefore, the search for new, more effective, and safe drugs is a priority (4, 5).

*Leishmania* parasites reside inside macrophages (cells of the immune system), where they differentiate from promastigotes to amastigotes and multiply, causing cell rupture and afterward invading other macrophages, which contributes to the disease progression. Diagnosis of this disease usually involves direct microscopic examination of tissue or fluid samples to detect the presence of *Leishmania* parasites (6).

High-content screening (HCS) systems accelerate the process of drug discovery through rapid *in vitro* screenings of compound libraries in search of anti-leishmanial medicines (7). However, many laboratories carrying out studies for the development of anti-leishmanial drugs do not have easy access to HCS microscopes or specific proprietary software due to their high cost. They need to use traditional methods, such as manually counting intracellular parasites after Giemsa staining, which is a standard and cheap staining method commonly used in laboratories in developing countries. Nevertheless, this is a time-consuming, laborious, and subjective task and is prone to errors. Therefore, the development of an automated method for the detection of *Leishmania* parasites in microscopy images can be an alternative to speed up the parasite counting process in a large number of images in many laboratories that do not have this type of technology.

This article presents an approach to assist in the detection and quantification of macrophages and intracellular amastigotes of *Leishmania* parasites in images of Giemsa-stained slide fields using image processing techniques, and is organized as follows: the following section (Section 2) presents an overview of the related literature. Section 3 describes the images used in this study and the proposed method. Section 4 shows the different performance metrics. Section 5 discusses the results obtained. Finally, Section 6 presents the conclusions of the study.

## 2 Related work

This section presents the main studies related to the automatic detection of intracellular amastigotes of *Leishmania* parasites in microscopy images, showing the methods used, image databases, results obtained, and main limitations. In addition, some studies devoted to processing images of other types of parasites, such as *Trypanosoma cruzi*, are analyzed due to their relevance to the subject addressed in this work.

Noguera et al. (8) proposed a semi-automatic approach for counting *T. cruzi* amastigotes in Giemsa-stained images using the marker-controlled watershed transform as a segmentation technique and shape features like area and compactness to identify amastigotes from other cells. They used 100 images and obtained the following values: a precision of 93.71% ± 11.11%, a recall of 85.63% ± 10.63%, and an accuracy of 84.62% ± 9.96%.

de Souza Relli et al. (9) proposed a method for automating the counting of *T. cruzi* amastigotes in Giemsa-stained images. First, a morphological filter removes the complex image background; second, the Fuzzy C-means algorithm is used to segment collections; third, threshold processing is carried out to preserve infected cells; and finally, amastigotes are processed by morphological granulometry and filtered by average. They used the same images as Noguera et al. (8), but composed of 40 images, and reported similar performance rates.

Yazdanparast et al. (10) developed an open-source software called INsPECT to automate the infection-level measurement of *Leishmania* (intracellular) parasites using DNA fluorescent images. Their approaches use some morphological operations and a method called threshold for images with a decreasing probability density function. This study used fluorescent images and the article does not provide an evaluation of the method in terms of the standard measures of effectiveness (sensitivity, F1, etc.) that would facilitate a comparison with other approaches.

Neves et al. (11) proposed a method for counting macrophages and parasites in fluorescent images of *Leishmania*-infected macrophages based on blob detection, clustering, and separation using concave regions of the cells’ contours. They used 24 images and achieved a precision of 81.55% ± 1.09, a recall of 87.62 ± 0.93, and an F-measure of 84.48 ± 0.60 in the parasite detection, achieving a better performance than those reported by Leal et al. (12) and Nogueira (13).

Gomes-Alves et al. (7) proposed an automated protocol for the quantification of the intracellular form of *Leishmania* spp in fluorescence images. This protocol was designed to be used in two image analysis platforms, IN Cell Investigator Developer Toolbox (commercial) and the free open-source Cell Profiler, and was made using classical algorithms. The results provide the total number of macrophages and parasites, the number of infected macrophages, and the number of parasites per infected macrophage. Moraes and Alcântara (14) proposed another protocol for the quantification of the parasite loads of *Leishmania* parasites in fluorescence images. The technique can detect and quantify intracellular amastigotes, obtaining the total number of cells, ratio of infected cells, total number of parasites, and number of parasites per infected cells.

These last six studies analyzed fluorescence microscopy images; however, work with optical microscopy images, which is what we used, has been less addressed and is more accessible for developing countries. Other methods for segmenting evolutionary forms of visceral leishmaniasis in microscopic blood smears are shown in Farahi et al. (15), Salazar et al. (16), and Isaza-Jaimes et al. (17). The first two studies do not provide evaluations of their effectiveness in parasite detection and only express the results in terms of segmentation quality. The third study reports a percentage of parasite recognition of approximately 80%. Salazar et al. (16) and Isaza-Jaimes et al. (17) used the same image dataset provided by Farahi et al. (15).

Górriz et al. (18) employed a deep learning approach using the U-Net model for the segmentation of *Leishmania* parasites and classified them into promastigotes, amastigotes, and adhered parasites. Thirty-seven images were used to train the algorithm. An evaluation of this method in terms of Dice score, precision, recall, and F1-score resulted in percentages of 77.7%, 75.7%, 82.3%, and 77.7%, respectively, for the amastigote class.

Another recent study regarding the automatic counting of macrophages and amastigotes is presented by Coelho et al. (19). This article illustrates quite well what needs to be done but does not provide a statistical analysis to support the results.

de Araújo Gonçalves et al. (20) provided a comprehensive survey of computer vision methods for detecting visceral leishmaniasis in humans. They recognized the lack of image databases, the scarce use of deep learning techniques, and that the methodologies that use the segmentation procedure perform better in terms of accuracy.

de Araújo Gonçalves et al. (21) proposed two different methodologies that perform an automatic classification of images as either positive or negative for visceral leishmaniasis in humans. The first method used a convolutional neural network (CNN) based on LeNet and trained from scratch and the second one used a feature extraction with a pretrained CNN and three classic classifiers [random forest (RF), support vector machine (SVM), and XGBoost]. They achieved an accuracy of 78.7%, a precision of 94.1%, a recall of 64.0%, and an F1-score of 99.2% in their best classification results.

Finally, the same author [Gonçalves et al. (22)] proposed a deep learning approach using the U-Net model for the segmentation of visceral *Leishmania* (VL) parasites in images from bone marrow, precisely indicating the location of the amastigotes in the image. In the detection of VL parasites, a Dice index of 80.4% was obtained, as well as an intersection over union (IoU) of 75.2%, an accuracy of 99.1%, a precision of 81.5%, a sensitivity of 72.2%, and a specificity of 99.6%.

The development of deep learning techniques, such as convolutional neural networks, has become an active research topic in the field of medical image analysis (23). However, training deep networks requires a large number of annotated image databases and a lot of computing resources, which is not currently feasible in developing countries.

Some limitations of the studies presented in the state of the art are related to the image databases used, which are mostly private. It is important to stress the fact that currently the lack of a public standard database of Giemsa-stained light microscope images for this kind of study constitutes a limitation when comparing different methods, and this should be considered in the analysis of results.

This study presents an automatic approach for the segmentation and quantification of macrophages and intracellular amastigotes of *Leishmania* parasites from Giemsa-stained images of mice peritoneal macrophage samples using image processing techniques based on algorithms that do not pose high demands to the computer facilities needed, which might be a favorable characteristic in many places where applications like this are needed. The proposed technique focuses on the separation of overlapping amastigotes to improve the counting process. The research reported included the testing of standard classifiers and sets of features to discriminate amastigotes and artifacts that might be counted as false positives.

## 3 Materials and methods

**Figure 1:**
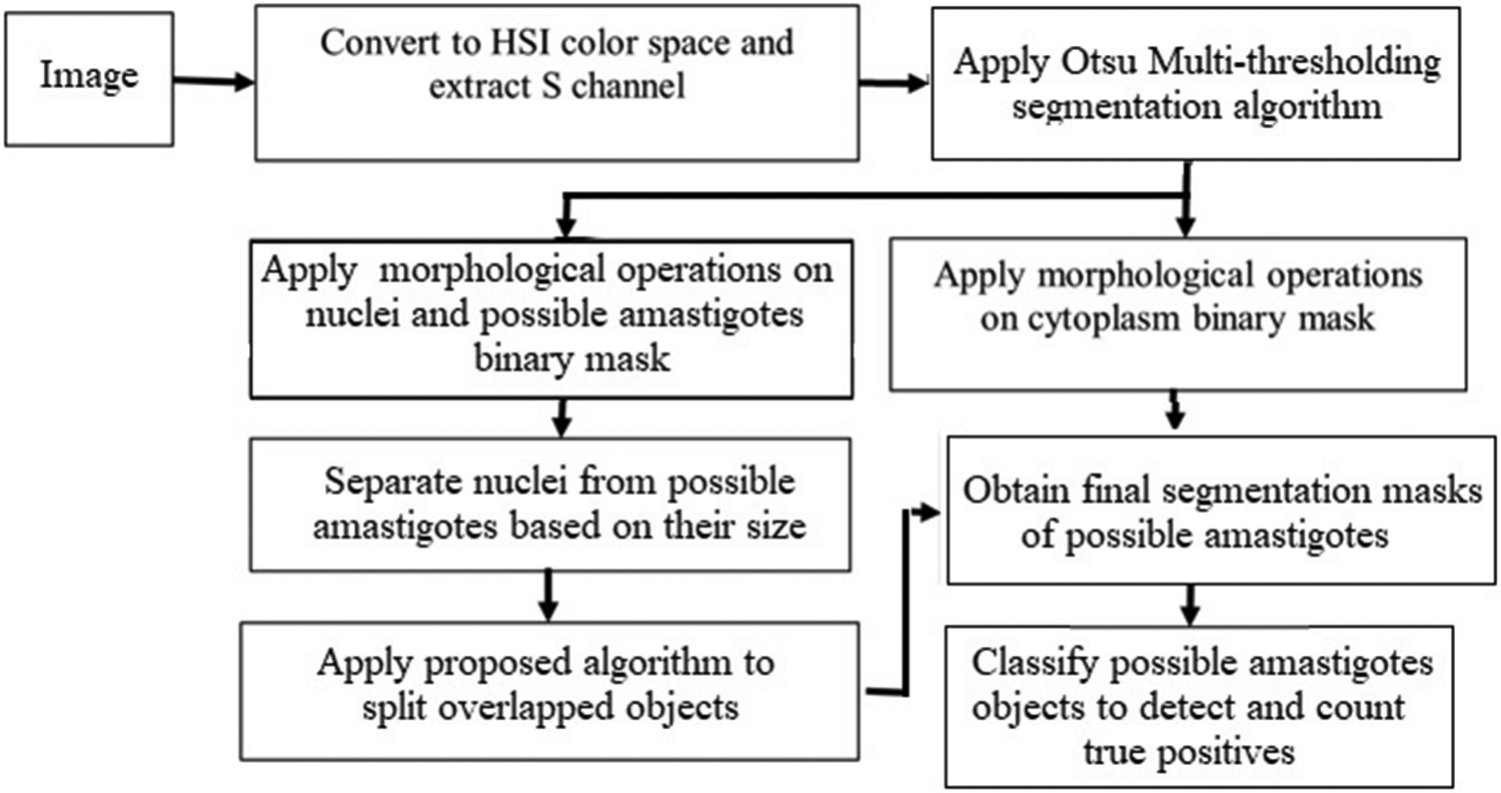
Block diagram showing the main steps in the proposed approach.

This section presents (1) the proposed methodology for the segmentation of object candidates to be classified as the amastigote form of the *Leishmania* parasite in macrophages, and (2) the classification process that completes the detection of amastigotes as true positives. Figure 1 shows a flow chart representing the main steps in the proposed approach.

### 3.1 *Leishmania* parasite culture

The experimental assay was carried out using the following protocol: the activity on intracellular amastigotes was studied in 24-well culture plates with coverslips and Roswell Park Memorial Institute culture medium supplemented with 10% fetal bovine serum and antibiotics (200 u/ml penicillin and 200 µg/ml streptomycin). A cellular suspension (10^5^ cell/ml) was added to each well. After 2 h at 33°C and 5% CO_2_, unadhered cells were eliminated and adhered macrophages were infected with stationary phase promastigotes at a 4:1 parasite/macrophage ratio. After 4 h incubation, the culture medium was discarded to eliminate free promastigotes. Then, fresh culture medium and the test compounds were added to reach final concentrations. After 48 h, the coverslips were removed and the cultures developed on them were fixed with methanol and stained with Giemsa.

### 3.2 Image acquisition

**Figure 2:**
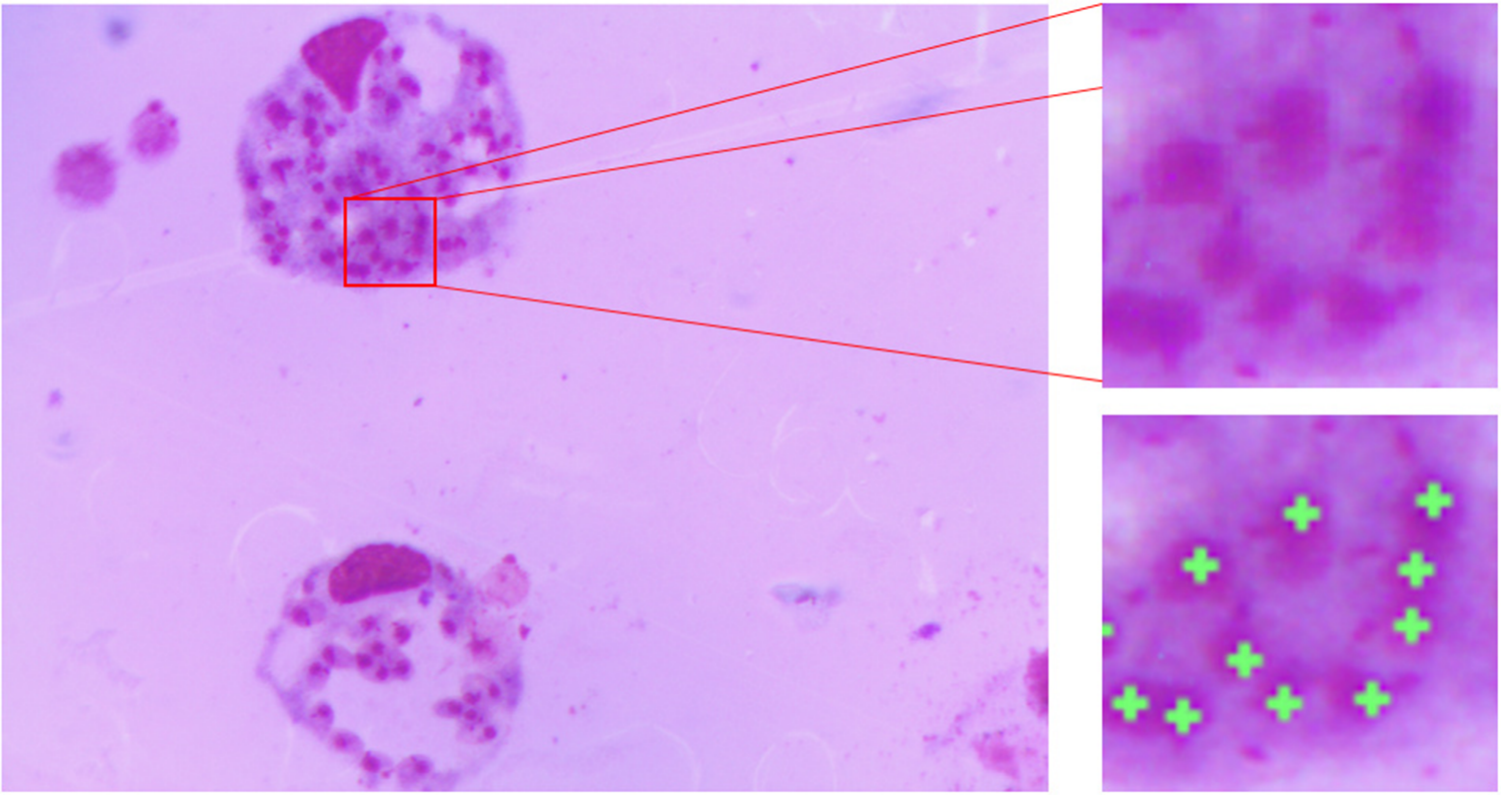
Giemsa stain image showing the amastigote form of *Leishmania* in which clumped amastigotes are magnified and the green crosses represent the manually annotated ground truth in the amastigote locations.

The image database was prepared at the Center for the Study of Chemical Bioactives (CBQ) at Universidad Central “Marta Abreu” de Las Villas (UCLV). The samples were prepared from *in vitro* peritoneal macrophages of BALB/c mice experimentally infected with *Leishmania amazonensis* and stained with Giemsa. The images were acquired with an Accu-Scope 3015 light microscope under a 100× objective (which combined with the camera lens results in a 50× optical magnification) with oil immersion and equipped with a 3.2-megapixel (MP) UCMOS03100KPA digital camera. Images were stored in a tagged image file format (tiff) with a resolution of 2,048 × 1,536 pixels. In this study, 46 images were captured. The ground truth images were prepared with the help of two experts from CBQ, who manually marked the locations of the amastigotes in each image. Figure 2 shows an image of macrophages infected with *Leishmania* parasites in which clumped amastigotes are magnified for better visibility. The green crosses in the locations of the amastigotes represent the ground truth annotations made by the specialist.

### 3.3 Pre-processing

**Figure 3:**
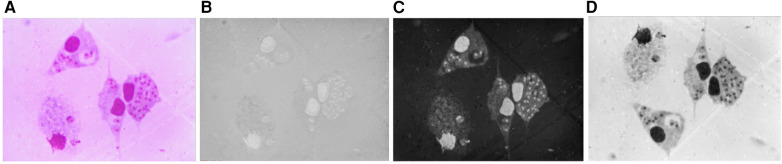
**(A)** Original image. **(B)** H component. **(C)** S component. **(D)** I component.

The pre-processing stage is an important step in improving the quality of the images. First, the original image was converted from red, green, and blue (RGB) to the hue, saturation, and intensity (HSI) color space. The HSI color space corresponds closely with the way humans describe and interpret color and also has the advantage that it decouples the color and grayscale information in an image, making it suitable for many techniques implemented for grayscale images (24). Figure 3 shows (A) the original image and in (B), (C), and (D) the hue, saturation, and intensity components in the HSI color space, respectively. As shown in Figure 3C, the saturation (S) channel shows the purple color of nuclei and amastigotes as the most saturated regions, allowing them to be more clearly identified. Therefore, the S channel was chosen to segment these objects.

### 3.4 Segmentation

In this study, it was necessary to segment the following objects: the amastigotes and the nuclei and cytoplasm of macrophages. The S channel image was selected because it could show the differences between these parts clearly enough. Here, the nuclei and amastigotes appeared as the brightest region in the image; therefore, they could be deemed as belonging to a single class but could be differentiated by their dimensions afterward. The cytoplasm appeared as a slightly dark gray region and the background of the image was practically black. These image characteristics led us to propose a multilevel thresholding segmentation with two thresholds to separate these different regions into three classes.

**Figure 4:**
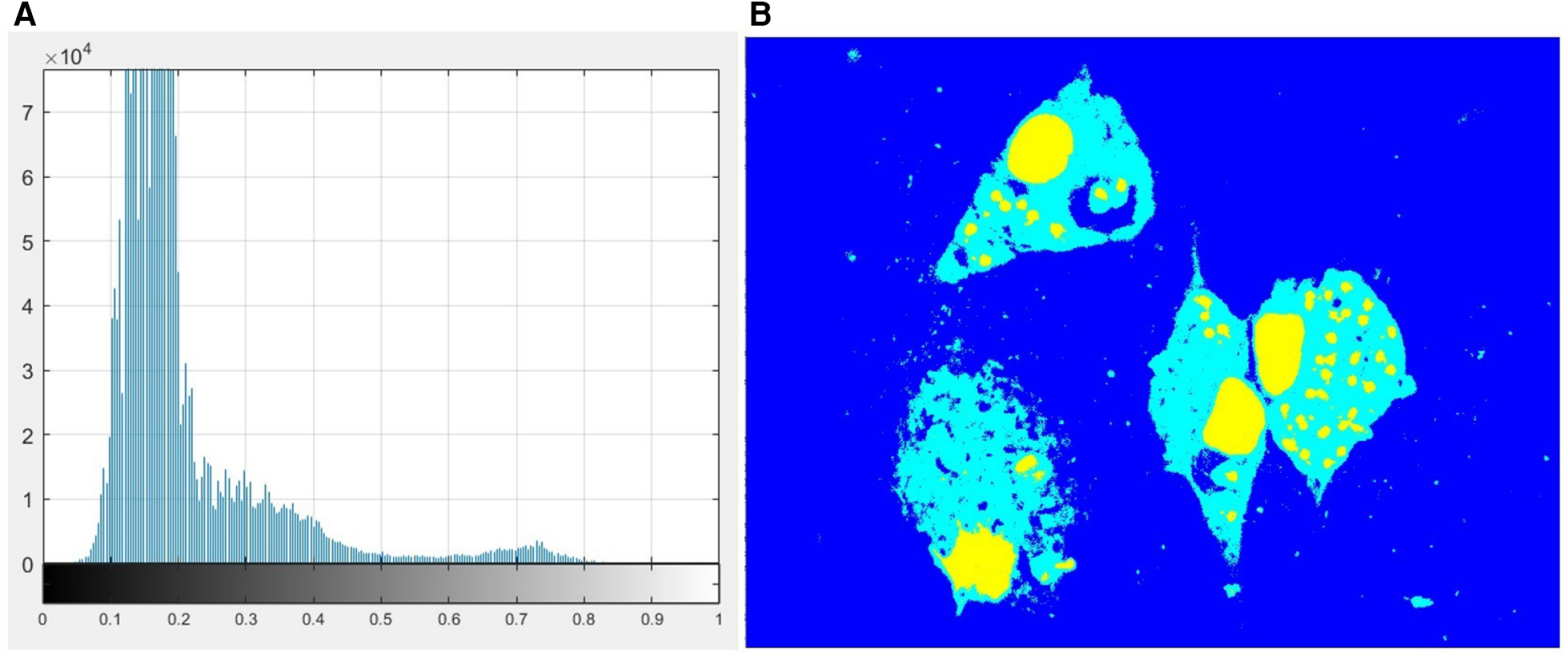
**(A)** Histogram of the image S channel. **(B)** Multilevel image thresholds using Otsu's method.

Otsu's method (25) is a widely used technique for image thresholding. This method found the optimal threshold by maximizing the between-class variance of pixel values, which effectively separates foreground and background regions. Thus, to find the thresholds that separated those three regions, we used the MATLAB built-in function called *multithresh*, which is an extension of the original Otsu method for multilevel thresholding. Figure 4A shows the histogram corresponding to the pixel distribution of the image Saturation channel and Figure 4B shows the result of applying this method on the S channel image. Once the segmented image was obtained, the next step was to separate these regions to obtain the binary mask corresponding to each class.

#### 3.4.1 Nuclei segmentation

**Figure 5:**
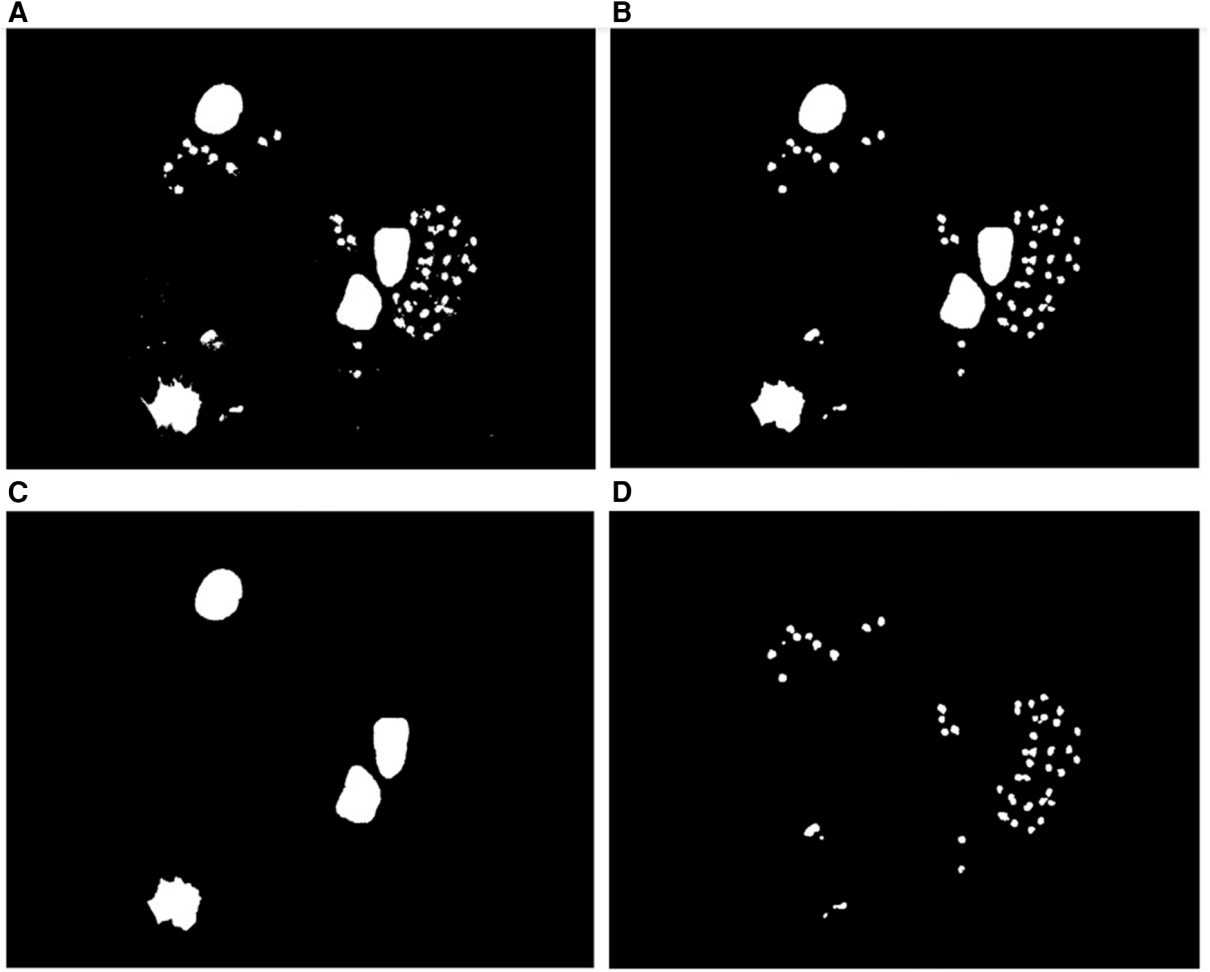
Nuclei segmentation. **(A)** Binary mask of the nuclei and parasite regions. **(B)** Binary mask of the parasite and nuclei regions after applying the morphological opening operation to fill holes and clear borders. **(C)** Binary mask of the nuclei regions. **(D)** Binary mask of the parasite regions.

After obtaining the binary mask of parasites and nuclei regions, morphological operations were performed on this mask to separate them and improve the segmentation of the parasites. First, the morphological opening operation was carried out with an approximately disk-shaped structuring element (SE) with a radius of 3 pixels to smooth the contours of nuclei and parasites, break narrow isthmuses, and eliminate thin protrusions. This radius size was defined based on the mean size of the amastigotes and on the resolution of the acquired image. Then, a morphological hole filling operation was applied to fill any “holes” caused by the thresholding process, and the objects that fell in the border of the image were removed. Finally, to separate the amastigotes from the nuclei, the size of the connected components (CCs) was analyzed by means of an area histogram; then, those objects with an area greater than 10,000 pixels were considered nuclei and the rest were considered as possible amastigotes, the binary masks of which would be refined in a further processing that will be described in a later section. This process is shown in Figure 5.

#### 3.4.2 Cytoplasm segmentation

To segment the cytoplasm regions, some morphological operations were applied to refine the initial binary mask of the cytoplasm obtained by Otsu's multi-thresholding algorithm. First, a morphological closing operation was applied with an approximately disk-shaped structuring element with a radius of 5 pixels to smooth the contour of the cytoplasm. Then, we filled the holes in the interior of the cytoplasm using a morphological hole filling operation. As the cytoplasm has a relatively large area, objects with an area less than 10,000 pixels (area of the nuclei) were removed using a morphological area opening operation.

**Figure 6:**
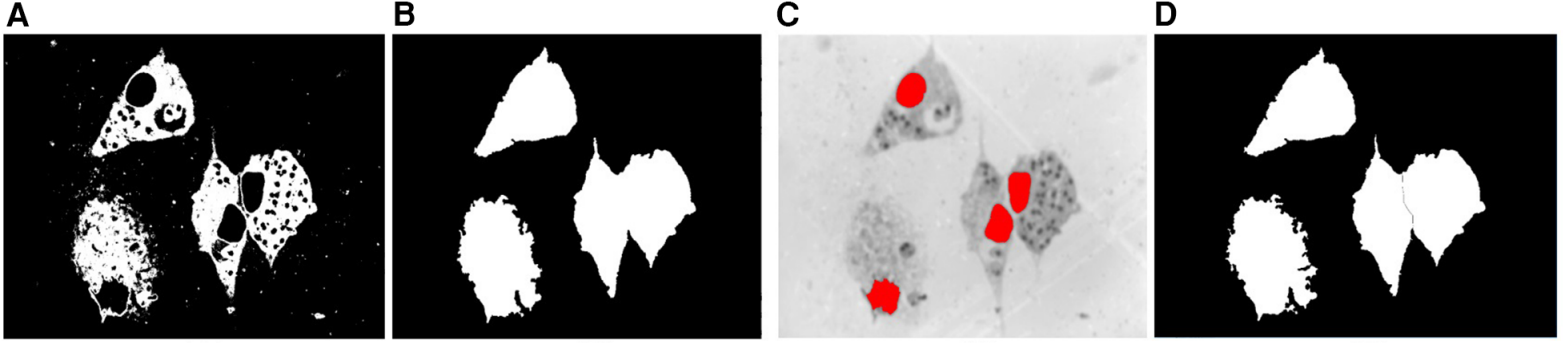
Cytoplasm segmentation. **(A)** Binary mask of the cytoplasm. **(B)** Binary mask after the morphological operations of hole filling and area opening. **(C)** Nuclei as internal markers on the complement of the Saturation image previously smoothed with a Gaussian filter. **(D)** Segmentation after applying the watershed transform.

The macrophages may have a different shape and size and we can also find touching macrophages that need to be separated. The use of the identified nuclei as markers for the watershed algorithm guarantees the exact number and location of the macrophages. To separate touching cytoplasm, we used the location of the nuclei obtained in the previous step as the seed for the watershed algorithm on the complement of the Saturation channel previously smoothed with a Gaussian filter. Figure 6 shows the separation process of two macrophages for the illustration of this procedure.

Then, each of the resulting connected components was analyzed because regions considered as artifacts may appear due to the stain used and the multilevel process. Therefore, we kept only those that, when superimposed with the nuclei mask obtained in the previous section, produced an intersection, which meant that this component was really the cytoplasm of a macrophage. Finally, as the image may contain dead macrophages, the cytoplasm that belonged to dead macrophages was eliminated, taking into account that the ratio between the area of the nucleus and the area of the cytoplasm is less than a threshold, which was determined experimentally with the value 0.65.

#### 3.4.3 Parasite segmentation

A macrophage was considered infected when it had at least one amastigote inside its cytoplasm. Then, we made an AND operation between the binary mask of the amastigotes obtained in Figure 5D with the binary mask of the cytoplasm obtained in Figure 6D, and the pixels that fell within the cytoplasm regions were considered possible amastigotes. However, there were some objects that significantly exceeded the mean size of the amastigotes, because some amastigotes were grouped or touching and needed to be separated. The set of processes described so far can be considered a first coarse classification based on the dimensions of the segmented structures, and the detected object candidates considered as amastigotes were subjected to a second stage involving the use of machine learning classifiers to find the true positives (amastigotes).

##### 3.4.3.1 Splitting overlapping amastigotes

One challenge in the segmentation of cells in microscopy images is to separate clustered, overlapped, or touching cells. After obtaining the binary mask of the possible amastigotes, the next important step in the proposed technique is the separation of the clusters into individual objects (possible amastigotes) to obtain a more accurate quantification. In this study, the marker-controlled watershed transform was used, based on one of the approaches proposed by Portuondo-Mallet et al. (26), which used morphological filtering and the weighted version of the external distance transform (EDT) [weighted EDT (WEDT)]. The proposed method includes two steps: (1) the detection of overlapping objects and (2) the separation of overlapping objects.

Step (1): The detection of overlapping possible amastigotes

**Figure 7:**
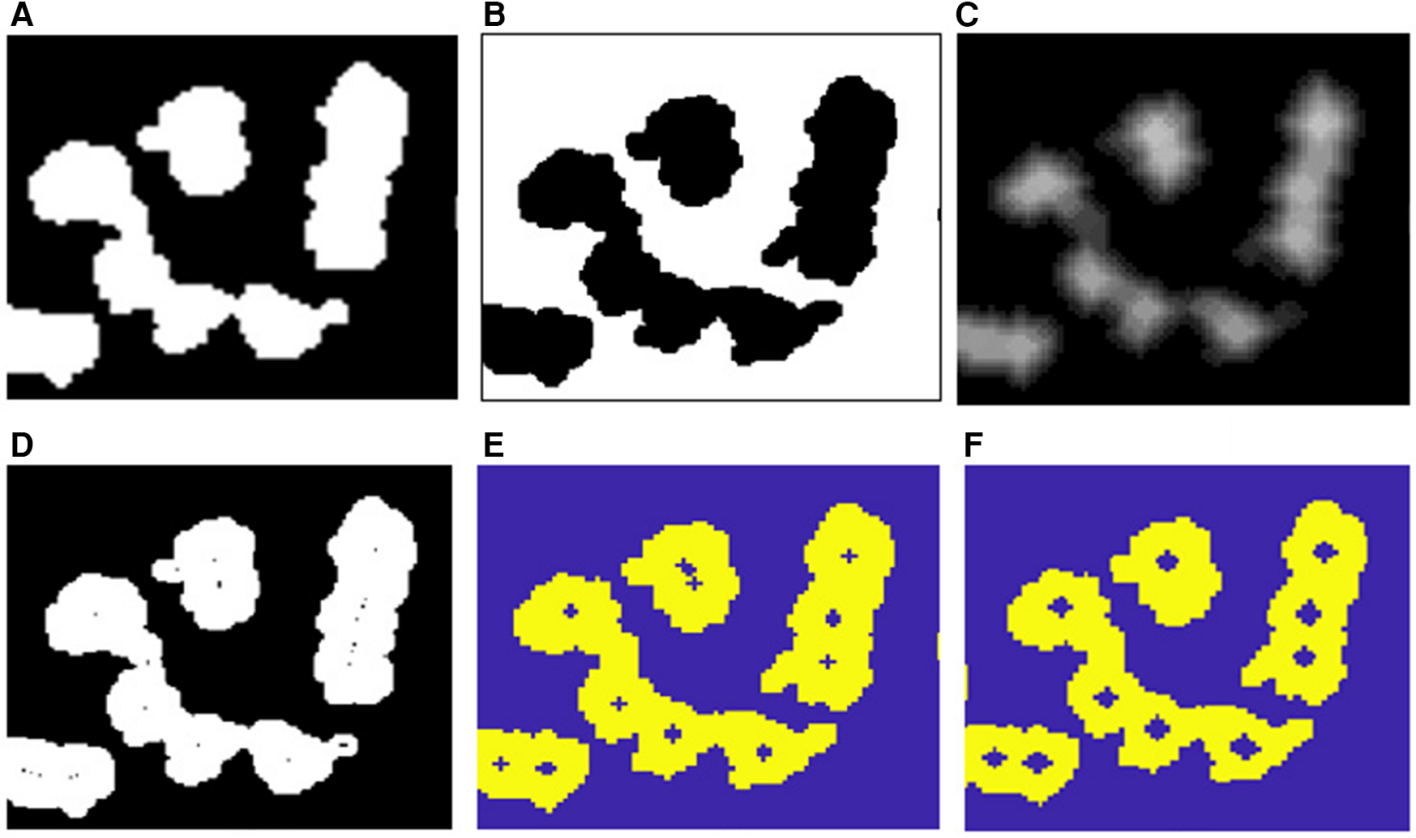
Detection of overlapped amastigotes. **(A)** Cropped binary image containing overlapped amastigotes. **(B)** Complement of the image in **(A)**. **(C)** Distance transform. **(D)** Regional maxima of the DT superimposed in **(A)**; notice the presence of spurious maxima. **(E**, **F)** Regional maxima after a two-stage open-close alternating sequential filter (internal markers) superimposed in **(A)**.

To split overlapped objects, first, the image of Figure 7A is complemented, as shown in Figure 7B, and then, its distance transform (DT) is computed, as illustrated in Figure 7C. The DT of a binary image can be defined as the distance from every pixel to the nearest non-zero-valued pixel (24). The result of DT is a grayscale image that shows its highest intensity in a point or patch, which is in general a regional maximum, located farthest from the background. Then, the obtained grayscale image is normalized to the range [0 1]. This image is called *Idt* here.

After calculating the DT, several spurious maxima may appear as shown in Figure 7D, which can lead to oversegmentation if used as markers for segmentation with the marker (controlled watershed transform). To remove the spurious maxima, a two-stage open-close alternating sequential filter (ASF) (27), using a disk structuring element *g* with radii 1 and 2 in the first and second filtering stages, respectively, was applied to the distance transform map shown in Figures 7E,F. The general expression for this filtering process is:

(1) $${\mathrm{ASF}}_{\mathrm{CO},g}^{2}(f)=(\;(((\;f\circ g)∙g)\circ 2g)∙2g)$$

for which in this case *f* is the *Idt* image. Here ° and ∙, respectively, represent morphological opening and closing. The resulting image is called *Idtco*. Then, we determined the regional maxima *Irm*, which became the inner markers for the watershed transform. Figure 7F shows the resulting maxima (inner markers) after applying this filter with radii 1 and 2, respectively, and superimposing it on Figure 7A.

Then, once the final regional maxima in *Irm* were obtained, each CC in Figure 7A was analyzed to determine how many of these regional maxima in *Irm* belong to each CC. This is carried out by computing a logical AND between these two images and putting the result of this intersection in *Irmcc*. If the number of labeled objects in *Irmcc* is greater than one, then the CC is classified as a cluster and should proceed to the splitting process, otherwise it is considered an isolated possible amastigote. In Figure 7F, we can observe that the connected components that have more than one regional maximum are clustered objects, and the connected components that only have one regional maximum are isolated possible amastigotes.

Step (2): Splitting overlapped objects (possible amastigotes).

The division of the overlapped possible amastigotes is carried out using the SplitClusterWEDT algorithm, which has been described in a previous study (26). This algorithm receives as input the CCs, the regional maxima of the cluster CC (*Irmcc*), and the DT image after the open-close filtering (*Idtco*). This procedure iterates for each regional maximum or inner marker in *Irmcc* and computes its WEDT, which is the EDT with its values divided (weighted) by a factor.

The EDT is defined as follows: consider the set *B* of pixels in the background (binary level 0) of the binary image. Then, for any point x∈B,EDT(B)(x) is the distance from *x* to the nearest pixel pertaining to a marker point (binary level 1), which in this case is the regional maximum that is being analyzed in the *Irmcc* image.

(2) $$\mathrm{EDT}(B)(x)=\min\{d(x,y)\;,\;y\in B^{C}\}$$

The factor used to divide the EDT of each regional maximum in each cluster is the value of the distance transform in the region occupied by each regional maximum. This factor contributes in obtaining a better location of the skeleton by influence zones (SKIZ) lines when segmenting clustered objects of different sizes.

**Figure 8:**
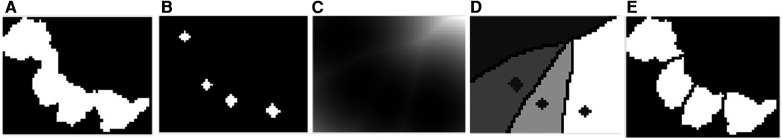
**(A)** Binary image of a cluster of amastigotes. **(B)** Regional maxima after morphological filtering. **(C)** WEDT. **(D)** Internal and external markers. **(E)** Split cluster.

Then, the algorithm computes the global WEDT map for the cluster, taking the minimum value of the WEDT in each point of the plane in which the image is located, which is calculated for each regional maximum in *Irmc*. Later, the watershed transform is calculated on this global WEDT map to obtain the SKIZ lines and segment the cluster CC. Figure 8 shows this procedure for one of the connected components featured in Figure 7A.

**Figure 9:**
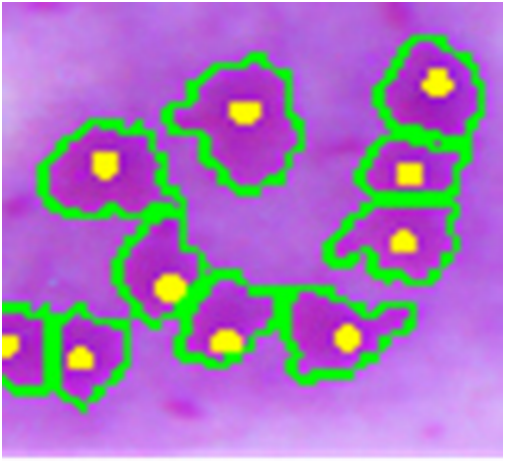
Amastigote contour detection (green) and reference ground truth marks (yellow).

Finally, the connected components with areas less than a third of the average area are considered artifacts and removed by an area open operation. This is necessary for the elimination of small objects that may interfere in the next steps. At this point, Figure 9 shows an example of the result of this segmentation, in which the green line is the amastigote contour and the yellow point is the reference ground truth mark.

#### 3.4.4 Final detection of amastigotes

So far, the described procedures were intended to detect the objects considered as possible amastigotes using mainly data related to the dimensions of the segmented objects. Given that the amastigotes exhibit some particular characteristics that differentiate them from possible spurious artifacts that cannot be distinguished only from data associated with their size and geometry, the experimental data taken from 46 annotated images, the processing of which resulted in 1,141 possible amastigotes from the first coarse classification described previously, were subjected to a feature extraction process.

##### 3.4.4.1 Feature extraction and selection

First, a set of 66 features associated with the possible amastigotes based on shape, texture, and intensity were extracted. Based on shape, eight features were extracted: area, perimeter, eccentricity, circularity, solidity, major axis length, minor axes length, and equivalent diameter. Based on texture, four features were extracted: contrast, correlation, energy, and homogeneity obtained from the gray-level co-occurrence matrix (GLCM) (28) for the cropped image of the bounding box of the segmented region in RGB converted to grayscale. Based on intensity, eight features were obtained for each color component of the RGB and HSI color space. These were mean, variance, standard deviation, skewness, kurtosis, smoothness, entropy, and the third moment. In addition, other intensity features were extracted: foreground–background contrast in red, foreground–background contrast in green, and foreground–background contrast in blue (29). Finally, another simple definition of contrast was extracted for each color component of the RGB color space:

$$K\;=\frac{G-G_{e}}{G_{e}}$$

where *G* and *G_e_* denote the mean gray value in the region and in the neighborhood, respectively.

In an attempt to optimize the dimensionality of the feature set, a subset of features was selected from the feature set using Waikato Environment for Knowledge Analysis (WEKA) version 3.8.6 (30). In this case, a wrapper method was selected (WrapperSubsetEval with the BestFirst method as a search method and J48 classifier). This selected 11 features, which are listed in Table 1. All the features were normalized with the normalize filter to the interval [0,1].

**Table 1:** Features used to classify possible amastigote objects.

No.	Description
1	Circularity
2	Major axis length
3	Minor axis length
4	Energy
5	K, Red
6	Mean, Hue
7	Kurtosis, saturation
8	Entropy, saturation
9	Standard deviation, red
10	Entropy, red
11	Kurtosis, green

##### 3.4.4.2 Classification

A set of three different classic classifiers, k-nearest neighbors (kNN), SVM, and RF, were compared to assess the results. In the case of kNN and SVM, various alternatives in their parameters (K = 1, 3 for kNN, and PolyKernel and Puk for SVM) were tested. The comparison amount of all the classifiers was performed using stratified tenfold cross validation, which ensures that each fold contains approximately the same proportions of different classes for all experiments as a measure of finding accuracy. Then, the results were analyzed to determine which of the schemes was (statistically) better than the other schemes. The indexes of effectiveness reported were the correctly classified instances (accuracy), incorrectly classified instances, TP rate, FP rate, F-measure, and kappa statistic. The experiments with classifiers were performed using WEKA and the results obtained by them are presented in the next section.

### 3.5 Evaluation

Results were assessed through comparisons between the automatically classified images and the corresponding manually annotated ones. True positive (TP) represents the number of manually labeled amastigotes correctly identified by the classifier algorithm, false negative (FN) denotes the number of manually labeled amastigotes not found by the algorithm, and false positive (FP) represents the number of amastigotes obtained by the algorithm without a corresponding manually labeled amastigote. The performance of amastigote detection was evaluated in terms of recall (also known as sensitivity), precision (also called the positive predictive value), and the F-measure, which were computed using Equations 3–5:

(3) $$\mathrm{recall}=\frac{\mathrm{TP}}{\left(\mathrm{TP}+\mathrm{FN}\right)}$$

(4) $$\mathrm{precision}=\frac{\mathrm{TP}}{\left(\mathrm{TP}+\mathrm{FP}\right)}$$

(5) $$F\text{-}\mathrm{measure}=\frac{2\times \mathrm{TP}}{\left(2\times \mathrm{TP}+\mathrm{FP}+\mathrm{FN}\right)}$$

## 4 Results

The processing was performed using MATLAB (R2019a version) on a computer with an Intel© Core © i7-6700 CPU @ 3.40–3.41 GHz, with 8 GB of RAM and a 64-bit Windows 10 Pro operating system. To evaluate the proposed segmentation and classification method, tests were performed on 46 images containing 157 macrophages. The 1,141 segmented regions (possible amastigotes) were composed of 975 amastigotes and 166 non-amastigotes based on the annotation made by the expert.

**Figure 10:**
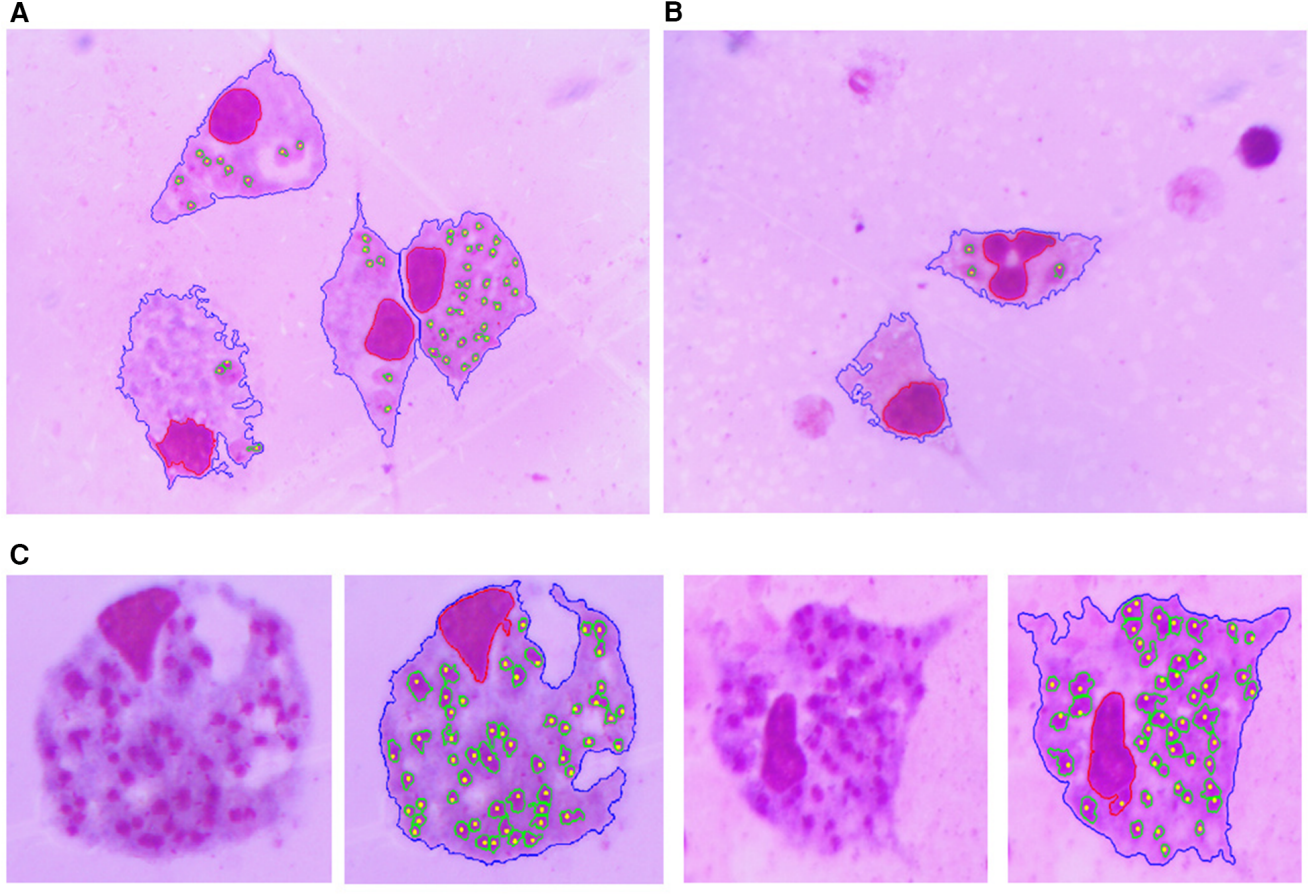
Segmentation result. The blue line is the outline of the macrophage, the red line is the nucleus contour, the green lines are the contours of the amastigotes and the yellow points are the reference ground truth marks. **(A)** Two touching macrophages are correctly separated. **(B)** Some artifacts produced by staining and a dead macrophage are properly removed. **(C)** Result of the detection and evaluation of amastigotes inside the macrophages.

**Figure 11:**
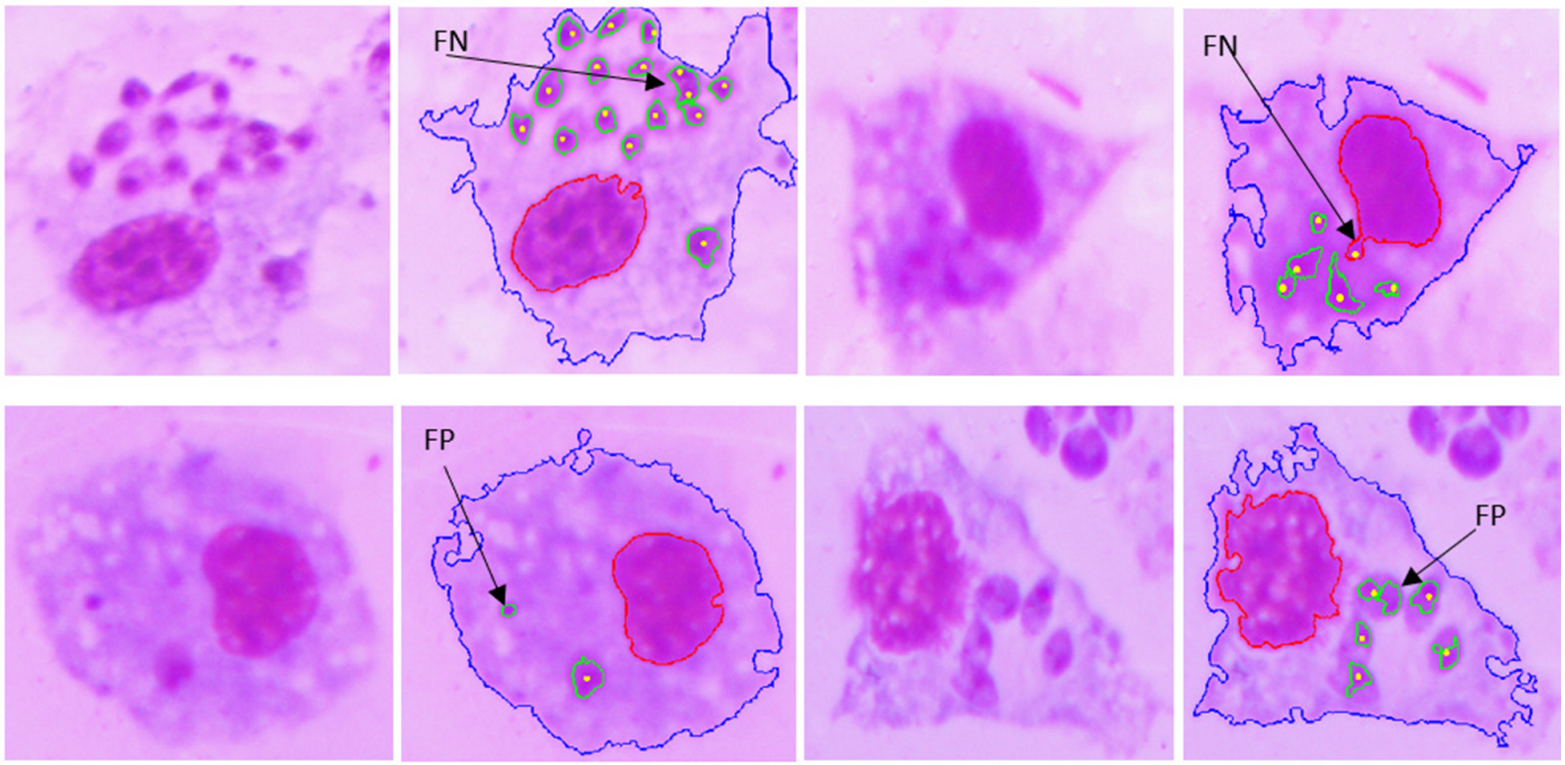
Examples of FNs and FPs obtained with the proposed approach.

Figures 10, 11 show examples of the segmentation results, in which the blue line is the outline of the macrophage, the red line is the nucleus contour, the green lines are the amastigote contours, and the yellow points are the reference ground truth marks for evaluation purposes. Figure 10A shows two touching macrophages that were correctly separated. In Figure 10B, some artifacts produced by staining and a dead macrophage are properly removed. Figure 10C shows the result of the segmentation of amastigotes inside the macrophages, with the annotation made by the specialist. A significant cause of a FN is shown in Figure 11; it was possible that the amastigotes were very close to each other or to the macrophage nucleus and the algorithm could not separate them, resulting in one FN.

The processing using classifiers is intended to improve the segmentation stage mainly in the reduction of FPs. The results of the classification process were obtained using WEKA Explorer and WEKA Experimenter with the segmented regions acquired in the segmentation phase.

The classification results acquired using the 11 features obtained using the WrapperSubsetEval feature selection method (with the BestFirst method, as a search method and J48 classifier) with the segmented regions are shown in Table 2. In this case, the performance measures used in a tenfold cross-validation experiment were the correctly classified instances (accuracy), incorrectly classified instances, TP rate, FP rate, F-measure, and kappa statistic. Notice that the best performance was obtained by the random forest classifier, with an accuracy of 93.3%.

**Table 2:** Weighted average of the classifiers used to detect the amastigotes from the set of possible amastigotes using the 11 selected features and tenfold cross validation.

Classifier	Correctly classified instance % (accuracy)	Incorrectly classified instances (%)	TP rate	FP rate	Precision	Recall	F-measure	Kappa statistic
kNN, K = 1	90.3593	9.6407	0.904	0.371	0.897	0.904	0.899	0.5786
kNN, K = 3	92.1122	7.8878	0.921	0.408	0.919	0.921	0.912	0.6178
SVM, PolyKernel	91.411	8.589	0.914	0.479	0.916	0.914	0.900	0.5572
SVM, Puk	92.4628	7.5372	0.925	0.403	0.924	0.925	0.916	0.6326
RF	**93** **.** **3392**	6.6608	0.933	0.306	0.930	0.933	0.929	0.7007

The bold represents the best result for the accuracy.

WEKA Experimenter allows more than one classifier to be tested simultaneously to classify the dataset. We tested the dataset by running the tenfold cross-validation test mode. The corrected paired t-test mode was used to verify the performance of each classifier by comparing the accuracy. The test used a 0.05 two-tailed confidence level and the results obtained with this test showed that there were no statistically significant differences between kNN, K = 3 and SVM, Puk with respect to random forest, respectively.

From a total of 975 amastigotes, 958 were correctly detected by the random forest classifier, which obtained the best results among those tested, and only 17 amastigotes were not detected. In addition, 107 were correctly detected as non-amastigotes and only 59 were considered false positives, improving the result of the segmentation phase. Table 3 shows confusion matrices obtained for the algorithms random forest, kNN with K = 3, and SVM with Puk.

**Table 3:** Confusion matrices from the classification results for random forest, kNN, K = 3 and SVM, Puk classifiers.

RF, % accuracy = 93.3392				kNN, K = 3, % accuracy = 92.1122			SVM, Puk, % accuracy = 92.4628		
Classified as **→**		a	b		a	b		a	b
Amastigote	a	958	17	a	964	11	a	967	8
Non-amastigote	b	59	107	b	79	87	b	78	88

## 5 Discussion

One aspect of the algorithms proposed in this study that deserves some clarification is the use of some parameters that are hard coded, i.e., not calculated from the primary data. The value of the size of the structuring elements (SE) (in terms of, for example, the radii of the various SEs) used for morphological operations as: opening with a discrete disk-shaped SE to smooth the contour of nuclei, break narrow isthmuses and eliminate thin protrusions; closing to smooth the contour of the cytoplasm; hole filling and open-close ASF, is closely related to the dimensions in pixels of the structures present in the images. This can also be said for the case of the 10,000 pixels threshold used in the area opening when segmenting the cytoplasm. The size of objects in pixels is determined by the relationship between microscope magnification (equivalent to 50× here) and camera resolution in pixels (2,048 × 1,536 pixels, 3.2 MP), and this size can be proportionally adjusted when using different values of these parameters, and the heuristically obtained values used in this study do not necessarily constitute a lack of generality.

Another possible source of difference between the method used in this study and those that might be obtained with a different set of images is the possible variation in color tones that could appear due to the sample preparation process or the light source used in the microscope. These differences could be solved by using color constancy techniques in an image pre-processing stage. This topic will be addressed in the continuation of this research.

The results obtained by the proposed method were compared with other state-of-the art methods cited in the present article. It is worth mentioning that in the literature review carried out, we found that there are few published studies on this subject and the image databases used in these studies are private and almost all of the images were obtained by fluorescence microscopy. Therefore, despite the fact that these studies did not use the same image database and, moreover, some included images of different types (fluorescence microscopy instead of light microscopy) and different parasites (amastigotes of *T. cruzi* instead of *Leishmania*), the global results can provide an idea of how the values obtained in the experiments reported in this article compare with those obtained in other studies in this field. Table 4 shows the comparison of our best results obtained with the random forest classifier with those presented in the previous discussion on the state of the art.

**Table 4:** Performance of the proposed detection method compared with others reported in the literature.

Author	Microscopy technique	Parasite	R (%)	P (%)	F (%)
Noguera et al. (8)	Light	*T. cruzi*	85.63	93.71	89.49^a^
Neves et al. (11)	Fluorescence	*Leishmania*	87.62	81.55	84.48
de Souza Relli et al. (9)	Light	*T. cruzi*	78.39	84.83	75.44
Górriz et al. (18)	Light	*Leishmania*	82.3	75.7	77.7
de Araújo Gonçalves et al. (21)	Light	*Leishmania*	64.0	94.12	76.19
Gonçalves et al. (22)	Light	*Leishmania*	72.2	81.5	76.57^a^
**Proposed method**	Light	*Leishmania*	**93**.**3**	93.0	**92**.**9**

P, precision; R, recall; F, F-measure.

The bold represents the best results.

^a^ Not available in the original study but as R and P are known and F = 2PR/(P + R), this measure was calculated to complete this table.

The method proposed by Noguera et al. (8), although it differs in the type of parasite, is similar to ours as they performed detection and counting of parasites and used the same type of image. However, they performed a semi-automatic method, whereas our method is fully automated and in terms of recall obtained better results.

The method proposed by de Souza Relli et al. (9) is similar to that of Noguera et al. (8) in the sense that it involves the detection and counting of the same type of parasite but with the difference that this is an automated method. They used the same images as Noguera et al. (8) and their performance measures were a little lower than those of Noguera et al. (8).

The method proposed by Neves et al. (11) allows the counting of macrophages and amastigotes of *Leishmania*; however, they used fluorescence microscopy images to make the process easier but when the objects are very close, separation may not be possible because important concave regions are not present.

The approach proposed by Górriz et al. (18), de Araújo Gonçalves et al. (21), and Gonçalves et al. (22) uses deep learning techniques. Górriz et al. (18) obtained promising results with just 37 images. de Araújo Gonçalves et al. (21) and Gonçalves et al. (22) obtained better results in terms of precision rather than recall. These are recent studies and it is significant to note their work in this field.

Training CNN models requires a large number of annotated image databases to obtain reliable results and a great deal of computing resource, which is not currently feasible in developing countries. Unfortunately, there are no publicly available databases of annotated images of these parasites in macrophages.

As can be seen, the results obtained in this study are favorable and perform better in recall and the F-measure than those presented in the state of the art. In addition, this method can provide good performance without a large image dataset or input image-size constraints, unlike deep learning methods.

## 6 Conclusion

In this study, an automated system for counting macrophages and intracellular amastigotes of *Leishmania* parasites in Giemsa-stained images using image processing techniques was proposed. The proposed approach is based on multilevel Otsu thresholding segmentation of the saturation component of the HSI color space, morphological operations, and the use of the watershed transform combined with the weighted external distance transform to split clusters or overlapping amastigotes. The results were evaluated in terms of sensitivity, precision, and the F-measure, which suggested a favorable effectiveness of the proposed method. The proposed method can assist in determining the *Leishmania* infection rate and makes the counting process in laboratories more expeditious.

The results presented here open the way to addressing some important extensions of this study. Among them we can mention the following: (1) quantifying a *Leishmania* sp. that does not live within enlarge parasitophorous vacuoles (PVs), instead of *L. amazonensis*, e.g., *L. braziliensis* and *Leishmania major*; (2) The classical quantification with this new approach using an *in vitro* incubation with a reference drug, such as amphotericin B, to obtain a table stating the differences in terms of intracellular numbers of amastigotes, the rate of infection, and the infectivity index, in addition to the time consumed for analysis; and (3) studying how this methodology would work when comparing different macrophages that are routinely used in *in vitro* assays (murine macrophage cell line vs. primary macrophages).

## Data Availability

The raw data supporting the conclusions of this article will be made available by the authors, without undue reservation.
